# Morphological and Chemical Diversity and Antioxidant Capacity of the Service Tree (*Sorbus domestica* L.) Fruits from Two Eco-Geographical Regions

**DOI:** 10.3390/plants10081691

**Published:** 2021-08-17

**Authors:** Igor Poljak, Nada Vahčić, Zlatko Liber, Katarina Tumpa, Valentino Pintar, Ivana Zegnal, Antonio Vidaković, Bernarda Valković, Davorin Kajba, Marilena Idžojtić

**Affiliations:** 1 Faculty of Forestry and Wood Technology, University of Zagreb, Svetošimunska Cesta 23, 10000 Zagreb, Croatia; ktumpa@sumfak.hr (K.T.); avidakovi@sumfak.hr (A.V.); dkajba@sumfak.hr (D.K.); midzojtic@sumfak.hr (M.I.); 2 Faculty of Food Technology and Biotechnology, University of Zagreb, Pierottijeva 6, 10000 Zagreb, Croatia; nvahcic@pbf.hr (N.V.); valkovic.bernarda@gmail.com (B.V.); 3 Department of Biology, Faculty of Science, University of Zagreb, Marulićev Trg 9a, 10000 Zagreb, Croatia; zlatko.liber@biol.pmf.hr; 4 Centre of Excellence for Biodiversity and Molecular Plant Breeding, Svetošimunska Cesta 25, 10000 Zagreb, Croatia; 5 Ministry of Economy and Sustainable Development, Institute for Environment and Nature, Nature Sector, Radnička Cesta 80, 10000 Zagreb, Croatia; valentino.pintar@gmail.com; 6 Croatian Forest Research Institute, Cvjetno Naselje 41, 10450 Jastrebarsko, Croatia; ivanaz@sumins.hr

**Keywords:** fruit tree species, population variability, morphometric analysis, chemical analysis, island populations, isolation by distance, isolation by environment, adaptation

## Abstract

Service tree, *Sorbus domestica* L., is a rare and neglected wild fruit tree species of southern and central Europe. Being distributed in different eco-geographical regions, with fragmented and low-density populations, *S*. *domestica* represents an interesting model case for investigating patterns of within- and between-population diversity at geographical and environmental scales. This study aimed to analyze the proximate composition, antioxidant activity, and morphometric fruit characteristics. We examined the diversity and population divergences of 49 *S*. *domestica* individuals originating from seven populations across continental and Mediterranean eco-geographical regions. In addition, tests of isolation by distance and environment were performed to detect the magnitude of divergence explained by geographic and environmental variables. Significant differences between the studied populations were found in almost all of the studied morphometric and chemical fruit characteristics. The studied service tree populations were characterized by high phenotypic variation despite the low number of trees per population. Model-based population structure analysis using morphometric and chemical fruit characteristics revealed three groups of service tree populations. We concluded that non-effective pollen and seed dispersal along with genetic drift and specific environmental factors resulted in a distinct phenotype with a specific chemical composition in the isolated island population. In addition, a pattern of isolation by the environment was revealed. We infer that morphological and chemical differences between the studied populations in the true service tree from different eco-geographical regions were mediated by adaptation to the specific environmental conditions.

## 1. Introduction

Forest fruit trees are species that play an important role in maintaining biodiversity, by enriching the gene pool of the forest ecosystem and with other trees and shrubs, ensuring the hardiness and health of the whole forest, as well as increasing the soil quality [1]. Due to their adaptability and resilience, the promotion of forest fruit tree species can be considered as one of the most promising efforts of climate change mitigation, and planting them is an efficient way of restoring degraded forests [2]. In addition, forest fruits represent an additional food source for wild animals, hence, being rich in antioxidants, minerals, and vitamins, they are recommended for human consumption as well [3]. As such, they have a large potential in the sustainable development of rural areas [4] and their usage and popularization are in line with sustainable resource management [5].

One of those forest fruit trees, almost forgotten in the previous decades, is the service tree. The service tree (*Sorbus domestica* L.) is an insect-pollinated, thermophile, and medium-sized deciduous fruit tree species of the Rosaceae family. It is found in southern and central Europe, with the center of distribution in the Balkan Peninsula, Italy, and southern France, sporadically appearing in North Africa and the Caucasus as well [6]. The extent of its original range is still unclear since it has been planted and sub-spontaneously spread since Roman times [7,8,9]. It is a light-demanding species, tolerant to different types of soil and occurring on a wide variety of sites [10,11]. Its distribution is scattered in two different eco-geographical regions, where it forms small and fragmented populations [12]. It can be found in a mild temperate climate on warm and south-facing slopes, and in the Mediterranean climate on dry to extremely dry, nutrient-poor sites. Although vigorous and relatively fast-growing, it is also a poor competitor, making it a rare species with low population density, with fruit-bearing trees being even scarcer.

Service tree truly does provide a number of services-high-quality wood, edible fruits, and health benefits, to name a few [13,14]. In addition, it is also a valuable species in ecosystems and landscapes, and it can be used as an ornamental tree in urban areas. Nevertheless, it is usually known as a fruit tree species. Its pear-shaped (f. *domestica* = f. *pyriformis* (Hayne) Rehder) or apple-shaped (f. *pomifera* (Hayne) Rehder, = f. *maliformis* (Kirchn. et J.Eichler) Hegi) fruits can be used for various culinary and medicinal purposes [15]. The fruits are pomes olive-brown or yellowish in color, flushed with red on the side facing the sun, often glaucous, with numerous large lenticels, maturing in September to October [16]. The weight of one pome is 15–32 g and its diameter is 1.2–3 (4.5) cm. Fruit flesh (enlarged fleshy hypanthium) is astringent and hard at maturity, edible after bletting by frost or in the state of incipient decay, with numerous sclereids. The fruit usually contains 1–6 (10) broadly obovoid, flattened, and reddish-brown seeds. The seeds are dispersed by birds and mammals [17]. The fruits can be eaten fresh or used to produce marmalades, juices, fruit wine, and to conserve apple cider. Although service tree fruits are nutrient-rich and suitable for processing, they are currently undervalued by the consumers and will need to gain in popularity to compete with the wide range of attractive and tasty fruits currently on the market.

Edible fruits characterizing this species make it an ideal model for the determination and further analysis of morphological and chemical diversity of populations. To date, only several studies of the morphological characteristics [18,19,20,21,22] and/or chemical composition [23,24,25,26,27] of the fruits have been carried out on the service tree. The fruit’s chemical composition was studied by different analyses including total phenols, flavonoids and tannins, macro- and micro-elements, and determination of antioxidant activity. However, to the best of our knowledge, until now no data have been published on the proximate composition in this species. The morphological variability of *S*. *domestica* fruit across a larger geographical range has not been studied to date either. In addition, several studies have been performed on *S*. *domestica,* including leaf morphometric analysis [28,29] and leaf antioxidant activity [30].

In this study, we combined morphological and chemical fruit traits to examine the diversity and divergence of seven service tree populations distributed across the Croatian continental and Mediterranean eco-geographical regions. In total, we included fruit samples from 49 trees. The small number of sampled trees was a direct result of the small density of populations [6,8,9,29], and the very low number of fruit-bearing trees. Our main objectives were to evaluate morphological diversity, fruit proximate composition, total phenols, antioxidants activity, and population structure, and to test the correlations between geographic, environmental, morphological, and chemical variation in *S*. *domestica*. Chemical differentiation was examined using ten chemical characteristics, and morphological variation was assessed for ten fruit traits. Finally, a variety of multivariate analyses and Mantel tests were used to examine the roles of geographic and environmental isolation in determining the morphological and chemical diversity in wild fruit tree species.

## 2. Results

### 2.1. Climate Differences among Sampling Sites

Nineteen bioclimatic variables were used to describe the environmental differences between seven studied populations (Table S1). The first principal component (PC1) explained 65.5% of the total variation and clearly separated the continental (P01 and P02) and Mediterranean (P03-P07) populations (Figure S1 and Table S2). The continental region is characterized by cold winters, with a more even distribution of rainfall throughout the year. The annual mean temperature is lower than that in the Mediterranean region, as well as the mean temperature of the coldest month. In contrast, the Mediterranean region features hot to very hot summers and mild winters. A distinctive feature of the Mediterranean region is the seasonality of rainfall distribution, with the majority of precipitation in the coldest quarter of the year.

### 2.2. Fruit Morphometric Characteristics

Based on all parameters of fruit size, the continental populations were shown to have larger fruits than the Mediterranean populations (Table 1, Figure S2). Fruit mass had a mean value of 7.32 g, and it was the second most variable trait, with a mean coefficient of variation of 36.04%. The least variable traits were fruit (FL) and seed (SL) length and fruit (MFW) and seed (SW) width.

Fruit width and fruit length ratio (FW/FL) values for the continental populations and Mediterranean population Istria were approximately 1.0, and for other south Mediterranean populations, 1.1. Although fruits of slightly oblong shape, e.g., pear-like circular or oval, characterized southern Mediterranean populations, and apple-like shape continental and north Mediterranean population Istria, both types of fruits were found in all studied populations. Fruit shape was a far less variable characteristic, with a mean CV of 10.17%.

Populations from the continental region had wider seeds than the populations from the Mediterranean eco-geographical region. The mean seed number (NS) varied from 1.25 in population Novi Vinodolski to 3.66 in population Brač, with an overall mean value of 1.88. Seed number was by far the most variable trait, with a CV of 60.06%. In general, Mediterranean populations were characterized by a larger number of smaller and more variable seeds.

All fruit and seed parameters were significantly different between individual trees and populations, except fruit width and fruit length ratio (FW/FL) and seed length (SL) (Table 2). The majority of tested parameters were characterized by having most of the variability linked to intra-population variability.

### 2.3. Proximate Analysis and Acidity

The analyzed service tree fruits were characterized by high water content and sugar levels and very low fat and acidity values (Table 3). Differences between the studied populations were confirmed for all studied proximate constitutes, except for acidity. The mean water content value was 65.14 g per 100 g dm. Values were relatively uniform, and the average coefficient of variation was low (CV = 6.70%). In general, Mediterranean populations had somewhat lower water content values than continental populations. Sugar content had a mean value of 49.36 g per 100 g dm and a coefficient of variation of 9.33%. The lowest values were found in population Brač (42.88 g per 100 g dm), and the highest in population Novi Vinodolski (55.60 g per 100 g dm). The mean protein value was 14.69 g per 100 g dm. Protein content was characterized by relatively high variability, with a mean coefficient of variation of 20.27%. Both ash and fat content were very low, with mean values of 2.12 and 0.72 g per 100 g dm, respectively. Both of these parameters were very variable, with mean coefficients of variation for fat of 19.52%, and ash content being the most variable parameter in general, with a coefficient of variation of 34.23%. Mediterranean populations were characterized by significantly higher fat content than the continental populations. Acidity values ranged from 0.64 to 0.74, with a mean value of 0.68. The coefficient of variations showed intermediate variability of data, with a mean value of 14.90% and a range of 5.24–28.20%. The cellulose content varied between the studied populations without a clear pattern. The average cellulose content was 6.24 g per 100 g dm, with a mean coefficient of variation of 27.95%. The population Brač was characterized by the highest cellulose content, 9.06 g per 100 g dm.

### 2.4. Total Phenolics and Antioxidant Capacity

Total phenolic content mean values ranged from 3.50 mg GAE/g dm to 12.10 mg GAE/g dm (Table 3). Continental populations have the highest values, 11.11 mg GAE/g dm (Psunj) and 12.10 mg GAE/g dm (Tounj), whereas island population Brač and population Split have the lowest, 3.50 mg GAE/g dm and 5.62 mg GAE/g dm, respectively. Populations Psunj and Brač have the highest coefficient of variation, 63.19%, and 57.44%, while population Tounj has the lowest variability of 16.80%, followed by population Istria, 38.23%.

Measured DPPH values ranged from 4.40 to 17.76%. Populations Istria (17.76%) and Split (13.76%) have the highest values, and Brač (11.70%) and Tounj (4.40%) have the lowest. Measured DPPH values were less variable than those of total phenolic content, with a coefficient of variation of 44.00%. Mean FRAP values ranged from 2.41 to 2.91 mmol Fe^2+^. The highest value was calculated for population Tounj, and the lowest for population Psunj.

### 2.5. Population Structure, Isolation by Distance, and Environment

**Figure 1:**
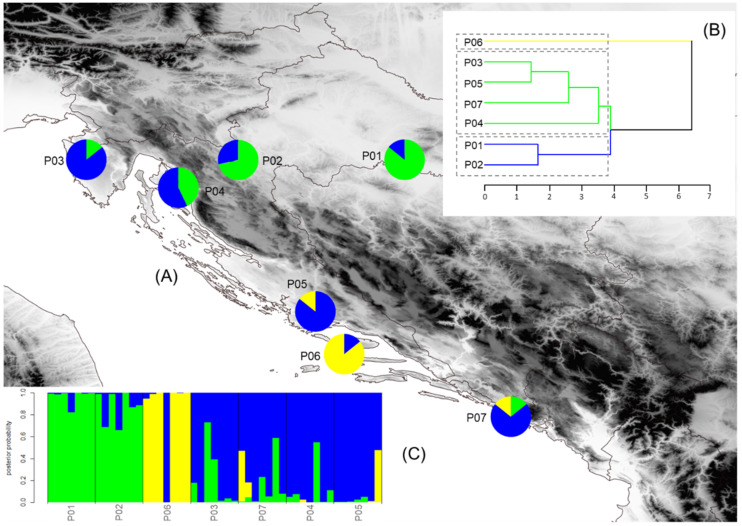
Results of the multivariate statistical methods for fruit and seed morphometric analysis and locations of the seven sampled *Sorbus domestica* populations. (**A**) Geographical distribution of three groups of populations detected from K-means clustering (the proportions of the ancestry of each population in each of the defined clusters are color-coded: cluster A– green, cluster B–yellow, cluster C–blue); (**B**) Hierarchical tree dendrogram; (**C**) Barplot with posterior probabilities of classification of each individual into each group from the results of the classification discriminant analysis. Acronyms of populations: P01—Psunj; P02—Tounj; P03—Istria; P04—Novi Vinodolski; P05—Split; P06—Brač; P07—Konavle.

**Figure 2:**
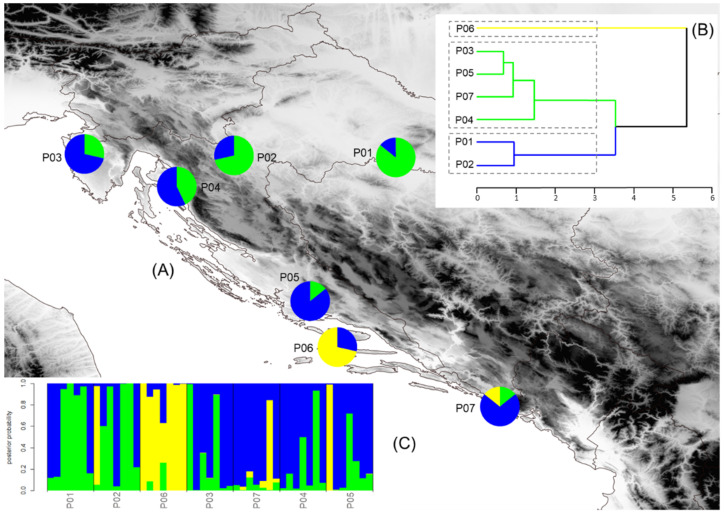
Results of the multivariate statistical methods for fruit chemical analysis and locations of the seven sampled *Sorbus domestica* populations. (**A**) Geographical distribution of three groups of populations detected from K-means clustering (the proportions of the ancestry of each population in each of the defined clusters are color-coded: cluster A– green, cluster B–yellow, cluster C–blue); (**B**) Hierarchical tree dendrogram; (**C**) Barplot with posterior probabilities of classification of each individual into each group from the results of the classification discriminant analysis. Acronyms of populations: P01—Psunj; P02—Tounj; P03—Istria; P04—Novi Vinodolski; P05—Split; P06—Brač; P07—Konavle.

**Figure 3:**
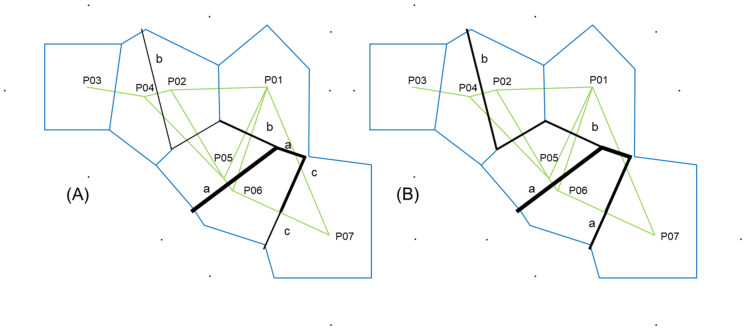
The boundaries obtained with Monmoniers’s maximum difference algorithm of Euclidean distances among populations revealed by program BARRIER. (**A**) The first three boundaries (a–c) for morphometric variation; and (**B**) the first two boundaries (a and b) for chemical variation. The thickness of barriers is related to their importance. Acronyms of populations: P01—Psunj; P02—Tounj; P03—Istria; P04—Novi Vinodolski; P05—Split; P06—Brač; P07—Konavle.

The structure of the seven service tree populations was inferred by the K-means clustering method. The most probable division was detected at K = 3, and the estimated population structure is shown in Figure 1A and Figure 2A. If the proportion of a certain population was equal to or higher than 0.70, it was assumed that the population belonged to one cluster, and if it was lower than 0.70, it was assumed that the population had a mixed origin. In both analyses, morphometric and chemical, we revealed the same geographical pattern. The samples from the populations Psunj and Tounj grouped into cluster A, the samples from island population Brač grouped into cluster B, and the samples from the coastal Mediterranean populations into cluster C. Only one population, P04 (Novi Vinodolski), was of mixed origin, with the dominant proportion from cluster C. The results obtained with the K-means clustering method were congruent with the results from the hierarchical clustering method (Figure 1B and Figure 2B) and Barrier software (Figure 3A,B). The existence of barriers was revealed between the continental and Mediterranean populations, as well as between the island population Brač and all other studied populations.

**Figure 4:**
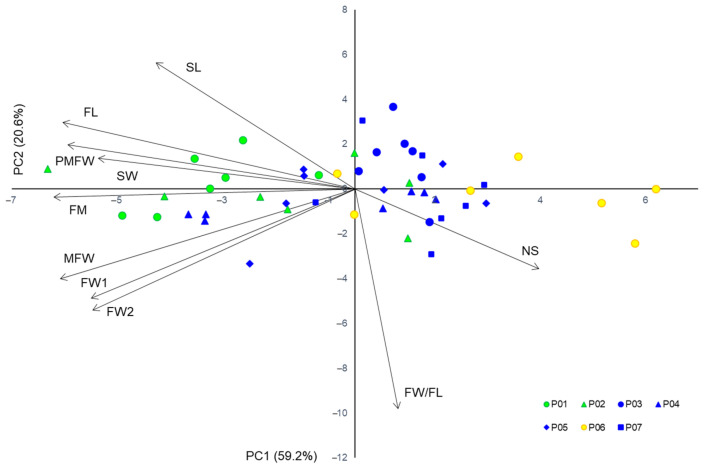
Biplot of the principal component analysis based on ten fruit and seed morphometric characteristics in studied *Sorbus domestica* populations. Acronyms of populations: P01 (Psunj), P02 (Tounj), P03 (Istria), P04 (Novi Vinodolski), P05 (Split), P06 (Brač), and P07 (Konavle). Each point represents a single tree, with a color corresponding to cluster from K-means clustering method and origin of population: continental populations—cluster A (green); island population Brač—cluster B (yellow); and coastal Mediterranean populations—cluster C (blue).

The first two components from a PC analysis of the phenotypic traits explained 59.2% and 20.6% of the total variation, respectively (Table S3 and Figure 4). PC1 was highly negatively correlated with the fruit size, and moderately positively with the seed number. On the other hand, PC2 was highly negatively correlated with the fruit shape, and moderately positively correlated with the seed size. Continental populations were associated with larger fruits with a smaller number of seeds, and Mediterranean populations with smaller fruits with a higher number of seeds.

**Figure 5:**
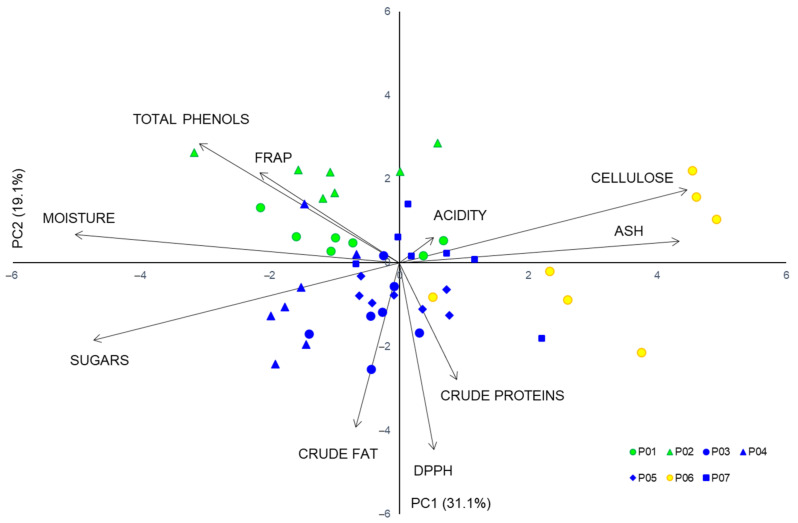
Biplot of the principal component analysis based on ten chemical traits in studied *Sorbus domestica* populations. Acronyms of populations: P01 (Psunj), P02 (Tounj), P03 (Istria), P04 (Novi Vinodolski), P05 (Split), P06 (Brač), and P07 (Konavle). Each point represents a single tree, with a color corresponding to cluster from K-means clustering method and origin of population: continental populations—cluster A (green); island population Brač—cluster B (yellow); and coastal Mediterranean populations—cluster C (blue).

PC analysis of all individuals showed that 50.2% of observed chemical variability was explained by the first two principal components (Table S4 and Figure 5). The first principal component (PC1) was strongly negatively correlated with the water and sugar content, and strongly positively with the ash and cellulose content. Strong negative correlations were observed between the second principal component (PC2) and the fat content and DPPH. Clear differences were revealed between the samples from the island Brač and other studied populations.

Discriminant analysis was performed to determine which of the morphological and chemical traits were the most useful for maximum discrimination between the three groups of service tree populations established by the K-means clustering method. Four out of nine morphological traits (number of seeds, fruit length, position of maximum fruit width, fruit width at 15% of fruit length), and four out of ten chemical characteristics (water, crude fat, cellulose, DPPH), were determined by stepwise discriminant analysis to be the best differentiating variables between the studied groups of service tree populations (Tables S5 and S6). The discriminant function based on morphometric and chemical traits showed a classification success of 87.75% and 97.96%, respectively (Figure 1C and Figure 2C). Overall, these results confirmed the usefulness of morphometric and chemical fruit traits in discrimination of the studied groups of service tree populations.

**Figure 6:**
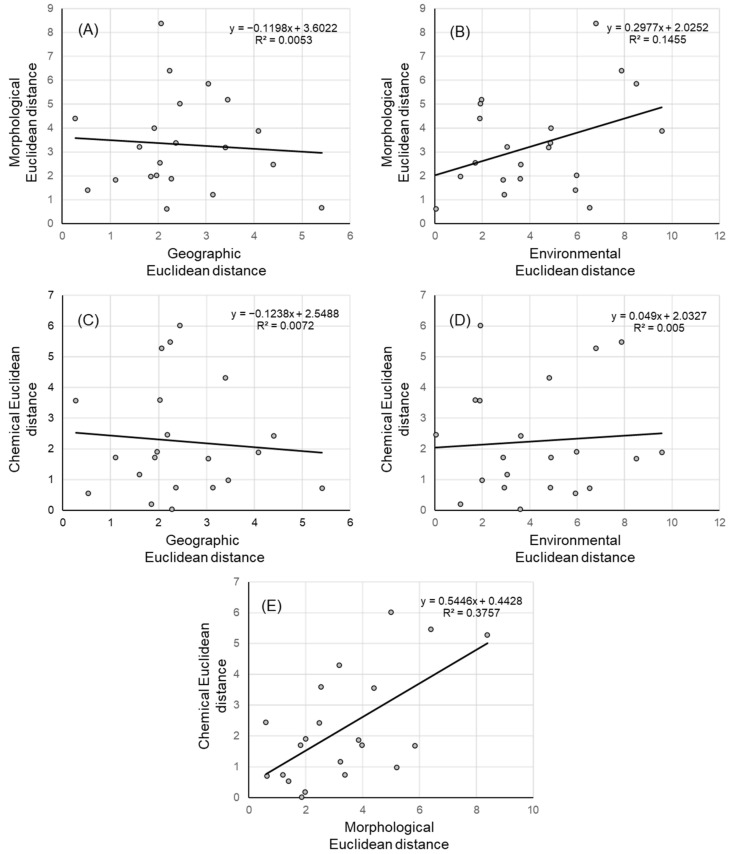
Isolation-by-distance and Isolation-by-environmental distance. Scatter plots of simple Mantel tests showing the relationships between (**A**) geographic and morphological distances (r = –0.0729, *p* = 0.58), (**B**) environmental and morphological distances (r = 0.3814, *p* = 0.05), (**C**) geographic and chemical distances (r = –0.0848, *p* = 0.47), (**D**) environmental and chemical distances (r = 0.0706, *p* = 0.37), and (**E**) morphological and chemical distances (r = 0.6129, *p* = 0.02).

Neither morphological nor chemical distances were related to geographical distances (Figure 6A,C). On the other hand, there was an overall relationship between pairwise phenotypic and environmental distances matrices, suggesting a pattern of isolation by environment (Figure 6B). Although we did not find significant correlations between pairwise chemical and environmental distances matrices (Figure 6D), several chemical traits were closely related to the environmental variables, i.e., Mediterranean populations were characterized by lower water and total phenolic contents, and higher fat contents in comparison with the continental populations. In addition, the Mantel test identified significant correlations between the morphological and chemical distance matrices (Figure 6E).

## 3. Discussion

### 3.1. Fruit Morphometric Characteristics

Our results clearly demonstrate a significant phenotypic diversity of the *S. domestica* fruits. The analyzed fruit characters had diversity levels similar to those reported for other tree species with similar life characteristics, especially regarding the non-continuous population structure, such as *Sorbus torminalis* (L.) Crantz [31,32], *Pyrus pyraster* (L.) Burgsd. [33,34], *Malus sylvestris* (L.) Mill. [35], *Prunus avium* (L.) L. [36,37] and various *Crataegus* L. taxa [38,39].

In general, populations from the continental eco-geographical region had larger fruits than populations from the Mediterranean region. The values of particular morphological traits found in our study did not differ considerably from the values reported in botany and dendrology textbooks [16,40,41,42,43] or morphometric studies [18,19,20,21,22]. However, the range reported by other authors was significantly wider than the range obtained in this study, i.e., the maximum values for fruit size, noted by other authors, were somewhat higher than those obtained in this research. In previously published papers, analyzed fruits were collected in wild populations and orchards. Our samples, however, were collected only from wild populations, which most likely meant less human interference and sub-optimal growing conditions. Interestingly, the levels of diversity in cultivated populations were similar to those in our study for the wild populations. High diversity in cultivated populations was probably preserved due to the fact that true service tree has never been subjected to selective breeding intensively, even though it has often been grown as a fruit tree [28]. According to Bignami [44], it was only propagated generatively by seeds for centuries and therefore preserved wide and underexploited variation in orchards as well as in natural populations.

The results of the analysis of variance (ANOVA) were in line with the expectations of high morphological variation within populations and low differentiation between populations, as observed in rowans [28,29,31] and other insect-pollinated and animal-dispersed tree species [45,46,47,48]. The relatively high level of among-tree variation within the populations is probably a result of both phenotypic plasticity to specific micro-environmental conditions experienced by each tree, and genetic differentiation among individual trees [28,29,49]. The within-population variation was of similar magnitude in all studied populations, except for the island population Brač. Even though isolated island populations could be expected to have significantly lower variability compared to the continental populations [50,51,52], island population Brač demonstrated the highest variability levels. This is more likely linked to the microsite features of its habitat than to genetic variability. Habitat conditions in which island samples were collected varied greatly, ranging from deeper soils to shallow, skeletal soils, stretched across varying altitudes. This indicates that phenotypic plasticity might be of greater importance with regard to microhabitat variation within service tree populations [53]. In addition, the island population Brač was morphologically the most distinguished population out of those analyzed. This population was characterized by having the smallest fruit with the highest number of seeds per fruit.

### 3.2. Proximate Composition

To the best of our knowledge, this is the first report on the proximate chemical composition of service tree fruits. Service tree fruits are sweet due to their high sugar content, are rich in proteins, and have a high content of cellulose, which acts as dietary fiber and as such can enrich a diet. We revealed that the parameters with the highest mean values were the least variable, e.g., water and sugar, and the most variable parameters were those with the lowest values-ash, cellulose, fat, and acidity. In comparison with the closely related rowan (*Sorbus aucuparia* L.), service tree fruits are characterized by lower water content, significantly higher levels of carbohydrates and cellulose, and similar, very low, fat content [54,55,56]. Results of the chemical composition analysis in this paper demonstrate great potential for further research on the specifics and benefits of service tree fruits and indicates potential future uses of fruits in both fresh and processed food supplements and products, like low-fat, protein-rich food.

This research has found significant differences between the populations in the analyzed chemical composition of service tree fruits. In general, the continental populations have somewhat higher water content and significantly lower fat values from cuticular wax. The differences between continental and Mediterranean populations are readily explicable by the specific ecological conditions in which populations grow. The Mediterranean populations are exposed to drought and extremely high temperatures, meaning their fruit need protection from the thicker cuticular wax, which acts as a shield against desiccation. It is reported that this protective layer in many plant species is a key evolutionary innovation in plants [57], and it has been implicated in protection mechanisms against different environmental stressors, such as excessive ultraviolet radiation, high temperatures, and salinity, low temperature during the vegetation season, etc. The current relationships between fruit chemical composition and environmental factors in *S. domestica* may be the result of long-term adaptation to different habitats.

Similar to morphological variability findings, intrapopulation variability was similar among all analyzed populations, with population Brač being the most variable. In addition, Brač was also the most specific population in terms of chemical composition. This island population was characterized by the lowest water content and highest ash and cellulose contents.

### 3.3. Total Phenolic Content and Antioxidant Activity

Several studies have demonstrated the importance of service tree fruits as a good source of different bioactive phytochemicals [26,41,58,59]. It was reported that those constitutes are mostly affected by geographic origin and genotype [26,60], climatic environment [61,62,63], and stage of maturity [64,65,66]. In general, it is well known that phenolic compounds represent a chemical interface between plants and the environment [67] and are under the influence of various environmental factors, including soil composition, temperature, and rainfall [68].

Significant differences in total phenols were found between the Mediterranean and continental populations, with higher values observed in the continental populations. This was somewhat opposite to our expectations since higher total phenolic content is expected in the sites where water deficit, along with high temperatures and increased UV radiation, causes stress in plants [69,70,71]. When subjected to environmental stress, plants respond by increasing the production of secondary metabolites, including phenolic compounds. The effect of moderate drought stress on the increased production of these compounds has been reported for several fruit species, namely *Punica granatum* L. [72,73,74], *Olea europaea* L. [75,76], and *Prunus dulcis* (Mill.) D.A. Webb [77]. Increased production of antioxidants and secondary metabolites, including phenols, neutralizes reactive oxygen and radicals [78]. However, prolonged drought leads to a reduction in the overall content of the metabolites due to greater growth reductions [71]. This could explain low phenolic values in the southernmost populations, island Brač, Split, and Konavle, since these populations grow in the Mediterranean climate, with prolonged drought and intense heat during the summer months. On the other hand, *S. domestica* is a tree species that prefers the Mediterranean climate. Therefore, in the continental eco-geographical region, where service tree grows in sub-optimal sites, adverse conditions that are more likely to become extreme could result in a higher total phenolic content in the fruits from continental populations. These adverse conditions can also be notable in pedoclimate, i.e., soil type. The continental populations grow on shallow, extremely dry, and basic soils, with carbonate, mostly marl-type parent material. The Mediterranean populations, although also facing shallow and dry conditions, grow on terra rossa of flysch or dolomite parent material with clayey texture, enabling better water retention and distribution, despite the increased amount of soil skeleton.

Measured DPPH values did not follow the same trend as total phenolic content. For example, population Tounj was characterized by the highest total phenolic content values but lowest DPPH, whereas the values for Psunj were very similar. DPPH free radical scavenging is a method for the evaluation of the antioxidant activity of all compounds in a sample, e.g., plant tissue. Natural antioxidants in plants include polyphenols (phenolic acids, flavonoids, anthocyanins, lignans, and stilbenes), carotenoids (xanthophylls and carotenes), and vitamins (vitamin E and C) [79]. Research on *S. domestica* has largely been focused on polyphenols, thus providing only a partial picture of all the components with antioxidative abilities and their relative contents. Apart from polyphenols, the biochemical composition of the fruits of *S. domestica* is characterized by 4 to 22% of carotenoids, 22.3 to 98.3% of ascorbic acid, as well as 1.8% of vitamin C [80]. It is therefore possible to assume phenols compromise only a small proportion of antioxidative compounds in *S. domestica* fruits, which would explain the variable data and relations between total phenolic content and DPPH assays. Furthermore, FRAP has not been shown to have a significant correlation with geographical or environmental distribution and is overall not significant as a parameter of distinction.

### 3.4. Population Structure, Isolation by Distance and Environment

Our results provide new insights into the morphological and chemical diversity and structure of service tree populations from two eco-geographical regions. In both cases, the studied populations were dived into three groups. The first group encompasses populations from the continental eco-geographical region, the second group samples from the island population Brač, and the third group populations from the coastal Mediterranean area. In both cases, island population Brač was specific, compared to other populations included in this research. Overall, these findings are consistent with the well-known fact that island populations are distinct because they have a unique array of traits/genes when compared with the mainland populations [81,82]. Scattered distribution and geographical isolation between those populations have probably resulted in a limited long-distance gene flow. Under such conditions, genetic drift may play a very important role in shaping the structure of the populations [28,83,84,85]. This microevolutionary process is especially pronounced in such low-density populations. Similar results were reported for leaf morphometric analysis of service tree populations [29]. Island populations were highly divergent in leaflets shape in comparison with adjacent mainland populations. However, it is reported that in temperate, insect-pollinated, and animal-dispersed tree species such as *S. domestica* [17,86,87] and *S. torminalis* [88,89,90], fragmented subpopulations are functionally connected by gene flow through both pollen and seed. This can explain connectivity between other coastal populations since they were in both cases classified in the same group. Also, we cannot exclude the possibility of human-mediated gene-flow as this species has been cultivated since Roman times [8,9]. Nevertheless, the clear pattern of morphological and chemical diversity indicates a natural origin of the studied populations.

The ‘mixed origin’ of the Novi Vinodolski population (P04) is of particular interest because our data indicate a certain gene flow between this population and the populations in the continental eco-geographical region. The Northern Adriatic region, stretching from Novi Vinodolski to Senj, has been linked to Central Croatia throughout history by trade and the exchange of goods. It is, therefore, safe to assume human-mediated transplanting and exchange of plant material happened between the continental population of Tounj and the Mediterranean population Novi Vinodolski, which would explain the mixed origin of the Novi Vinodolski population.

The tests for isolation-by-distance were not significant for the studied populations, and the morphological structure between the Mediterranean and continental populations was largely explained by the environmental conditions and fits the isolation-by-environment pattern. In addition, we revealed that water, crude fat, and total phenolic contents of the fruits were associated with environmental variables. Overall, our results suggest that populations from the continental and Mediterranean eco-geographical regions, despite their admixture in some cases, are isolated by the environment. It is well known that environmental heterogeneity can have a fundamental impact on phenotypic diversity [91,92,93]. In particular, different environments can result in spatially variable selection pressures, thereby contributing to phenotypic divergence among populations via phenotypic plasticity or local adaptation [92,94]. Lower annual mean temperatures accompanied by high precipitation during the warmest quarter in the continental eco-geographical region resulted in larger fruits with higher water content. On the other hand, higher annual temperatures, especially during the warmest quarter, resulted in smaller fruits with lower water content and higher fat content from cuticular wax. Overall, our results indicate that multiple morpho- and chemo-types, each having higher fitness in its native habitat than the others, are probably the result of local adaptation [95].

## 4. Materials and Methods

### 4.1. Plant Material and Study Area

The study encompassed two populations from the continental eco-geographical region (P01—Psunj, P02—Tounj), and five populations from the Mediterranean eco-geographical region (P03—Istria, P04—Novi Vinodolski; P05—Split; P06—Brač; P07—Konavle). In each population, samples were collected from seven trees, which were then used in morphometric and proximate analyses as well as studies of total phenolics and antioxidant activity. The small number of sampled trees is a direct result of the small density of populations [6,8,9,29], i.e., a small number of mature trees spread across large areas in the habitat. This fragmentation made collecting challenging and caused a smaller overall sample size.

To describe the environmental differences of the studied populations, for the principal component (PC) analysis and the calculation of environmental distances, 19 bioclimatic variables were obtained for each collection site.

### 4.2. Morphometric Analysis

In order to conduct the morphological analysis, 50 fruits were collected from each tree. Firstly, fruit mass (m) was determined with 0.1 g precision weighing. After weighing, fruits were cut longitudinally, and the dimensions were measured automatically using the WinFolia software [96]. The accuracy of measurements was 0.1 mm, and the following morphological characteristics were measured: fruit length (FL); fruit length, measured from the fruit base to the point of maximum fruit width (PMFW); maximum fruit width (MFW); and fruit width at 15% (FW1) and 85% (FW2) of fruit length. After the fruits were measured, seeds were extracted, the pulp removed, and the seed dimensions, i.e., length (SL) and width (SW), were measured automatically using the WinSEEDLE software [97]. In addition, the number of seeds per fruit (NS) was counted manually. Finally, ten morphological characters were examined in order to assess the variation within and between populations.

### 4.3. Proximate Analysis

Water, crude protein, crude fat, ash, cellulose, and sugar contents were determined according to the procedures established by the Association of Official Analytical Chemists (AOAC). All analyses were performed in duplicate. Water content in the samples was determined by a physical, indirect method, in which a sample of known mass was dried in an air dryer (Instrumentaria, Zagreb, Croatia) at 105 °C until a constant weight was achieved (4 h) [98]. Total mineral content was measured as ash content, which represented inorganic fruit compounds left after the organic matter was combusted [99]. The 4 g fruit samples, previously carbonized on open gas burners, were ignited in a muffle furnace (Nabertherm GmbH, Lilienthal, Germany) at 580 °C, until a constant weight was achieved, i.e., until uniform, light-grey ash with no black admixtures formed. The Kjeldahl method was employed to determine the total nitrogen content, in combination with a copper catalyst using the block digestion system Foss Tecator 6—1007 Digestor (Foss Tecator, Höganäs, Sweden) and the Foss Kjeltec™ 8100 Auto Distillation unit (Foss Tecator, Höganäs, Sweden). Crude protein content was obtained by multiplying total nitrogen by a conversion factor of 6.25 [100]. Total crude fat extraction was performed by the Soxhlet apparatus (Inkolab d.o.o., Zagreb, Croatia); medical-grade petroleum was used for extraction during 5 h [101]. Crude cellulose was determined by the method of Kürschner and Hanak [102]. Sugar content in the fruits was determined according to the AOAC 925.35 [103] method. Reducing sugars, glucose and fructose were determined using Fehling solution A, which was reduced to copper (I) oxide by the sugars, under specific conditions. Non-reducing sugar, e.g., sucrose, was firstly hydrolyzed down to reducing sugars, whose content was determined using Fehling solutions and used to calculate the total sugar content (total inverted sugars).

### 4.4. Determination of Total Phenolics and Antioxidant Capacity

#### 4.4.1. Fruit Extraction Procedure

Extraction was conducted according to Benvenuti et al. [104]. After the seeds were removed, fruits were finely chopped and 6 g (±0,01) were placed in a 250 mL Erlenmeyer flask. 20 mL of methanol (MeOH)/2% hydrochloric acid (HCl) (95:5) extraction solvent was added. The flask was sealed, wrapped in aluminum foil, and left for 1 h on the shaker. After 1 h on the shaker, extracts were filtered in a Büchner flask, using vacuum, and transferred into 50 mL volumetric flasks. The remaining extract in the Büchner flask was rinsed with a few mL of extraction solvent. Residue from the Büchner funnel was transferred into the same 250 mL Erlenmeyer flask and the procedure was repeated, resulting in a double volume of extract in the 50 mL volumetric flask. Flask was filled with extraction solvent up to the 50 mL mark and left refrigerated, at +4 °C, until further analysis. These extracts were used in determining the total phenolic content, the antioxidant capacity assay by DPPH method, as well as the reducing power assay using the FRAP method.

#### 4.4.2. Estimation of Total Phenolic Content Using the Folin-Ciocalteu Method

Total phenolic content in the samples was determined according to the Folin-Ciocalteau method [105]. This colorimetric method is based on the reduction of the phosphotungstic-phosphomolybdenum acid complex, creating a blue chromophore. Maximum absorption of the chromophores depends on the alkaline solution and the concentration of phenolic compounds, with a more intense blue color indicating higher absorption and concentration of antioxidants [106].

In a tube, 40 µL of diluted extract sample, 3.16 mL of distilled water, and 0.2 mL FC reagent were added and vortexed. After 3 min, 0.6 mL of aqueous sodium carbonate (20%) were added, the sample was vortexed again and placed in a water bath at 40 °C. After 30 min, the absorbance was read, at 765 nm. Total phenol concentration was calculated from the calibration curve, where gallic acid was the standard. Results were expressed as mg of gallic acid per g of dried fruit matter (seeds excluded) [24].

#### 4.4.3. Evaluation of Antioxidant Activity Using the DPPH Method

DPPH method is based on the reduction of stable radical 2,2-Diphenyil-picrylhydrazyl (DPPH*) in the presence of antioxidants. Due to its unpaired electron, DPPH* strongly absorbs in the spectrum of 517 nm [107]. Reduction causes stable, purple-colored DPPH* to change into yellow-colored DPPH-H [108,109,110]. Phenol compounds act as hydrogen donors, thus making this method suitable as an antioxidant assay.

A volume of 2 mL of diluted extract, 2 mL of methanol, and 1 mL of 0.5 mM DPPH methanolic solution were placed into a tube and shaken on a shaker. The blank was methanol without DPPH. Closed tubes were then kept in the dark, at room temperature, for 20 min. Absorbance was measured at a wavelength of 517 nm, against the blank of DPPH-free methanol. The DPPH* concentration in the reaction medium was calculated from the calibration curve for Trolox. Results were expressed as a percentage (%) of residual DPPH.

#### 4.4.4. Determination of FRAP

Ferric reducing antioxidant power (FRAP) assay is a method in which yellow-colored Fe^3+^-2,4,6-tripyridyl-s-triazine (TPTZ) complex is reduced to the blue-colored ferrous form, in a low pH-value medium [109]. The FRAP reagent was prepared by mixing 50 mL of acetate buffer with 5 mL of TPTZ and 5 mL of FeCl_3_ (10:1:1). For sample analysis, 240 µL of distilled water was mixed with 80 µL of appropriately diluted sample and 80 *µ*L of FRAP reagent. Blank was prepared in the same manner, substituting sample with extraction solution (methanol/HCl 2%). The mixture was left to stand for 5 min in a water bath, at 37 °C. Absorbance was measured at 595 nm. A calibration curve for FeSO_4_·7H_2_O was used to calculate FRAP values of the measured absorbances. Results were expressed as FRAP values, e.g., mmol Fe^2+^.

### 4.5. Statistical Analysis

Descriptive statistical parameters, arithmetic means, and coefficient of variation were calculated for the particular fruit morphometric characters and chemical composition for the studied populations. To assess the possibility of conducting multivariate statistical analyses and parametric tests, the symmetry, unimodality, and homoscedasticity of data were verified [111]. Assumptions of normality were checked using the Shapiro–Wilk test, and the assumption of homogeneity of variance using Levene’s test. A hierarchical analysis of variance (ANOVA) was performed to examine the partition of phenotypic variation between the studied populations and within populations. In addition, one-way ANOVA was used to test the differences in chemical composition between the studied populations. For each analysis, statistically significant differences between means were identified using the Fisher’s LSD multiple comparison test, at *p* ≤ 0.05. Descriptive statistics and ANOVA analyses were carried out using the program package STATISTICA [112].

To evaluate the correlation between multicharacter differences among populations, a Mantel test [113] was performed on the matrices of Euclidean distances. To assess isolation-by-distance and isolation-by-environment, morphological and chemical distance matrices were compared to the geographic and environmental distance matrices using the simple Mantel test [114,115]. To calculate the environmental distance matrix, climate data were obtained from the WorldClim 2 database with a spatial resolution close to a square km [116,117]. In addition, a simple Mantel test was performed between morphological and chemical distance matrices. The significance level was assessed after 10,000 permutations, and the Mantel test was performed with the R package “Vegan” [118].

To identify the divergence and structure of the studied populations, multivariate statistical methods were used [119]. Agglomerative hierarchical clustering algorithms were used to construct a tree diagram. Pairwise Euclidean distances were calculated, and cluster analysis was performed using the unweighted pair-group method with arithmetic mean (UPGMA). The K-means method was applied to detect structure and define the number of K-groups that best explained the morphological and chemical variation of populations (e.g., [49,120,121,122]). The eco-geographical structure of the studied populations was further analyzed using the Monmonier’ maximum difference algorithm, implemented in Barrier 2.2 software [123]. In addition, principal component (PC) analysis was used to calculate principal components across all individuals and all studied morphometric and chemical traits. The biplots were constructed by two principal components showing analyzed individuals and traits. To calculate the discriminatory power of characters among three groups of service tree populations established by the K-means clustering method, a canonical discriminant analysis was performed. The proportion of individuals correctly classified into the above-mentioned groups was determined using classificatory discriminant analyses [118,124]. The input data in multivariate statistical methods were previously standardized, i.e., standardization of characters to zero mean and unit standard deviation was performed prior to each multivariate analysis. The above statistical analyses were conducted using the statistical program R v.3.2.2 [125].

## 5. Conclusions

Our study showed high levels of phenotypic and chemical diversity for service tree populations despite its scattered nature and low-density populations. We revealed the existence of three morphologically and chemically distinct and well-defined groups of service tree populations. We suggest that those differences are the result of both neutral and adaptive microevolution processes. With regard to the island population Brač, non-effective pollen and seed dispersal, along with genetic drift and specific environmental factors, resulted in a distinct phenotype with specific chemical characteristics. On the other hand, environmental heterogeneity, i.e., differences between the Mediterranean and continental eco-geographical regions, resulted in an adaptive phenotypic and chemical evolution. Overall, the patterns found in this study confirmed that multiple evolutionary processes influence the morphological and chemical diversity and structure of the populations.

## Figures and Tables

**Table 1:** Arithmetic means (M) and coefficient of variations (CV/%) for fruit and seed morphometric characteristics. Different letters within one column denote statistically significant differences (*p* < 0.05) by Fisher’s LSD test. Acronyms of populations: P01—Psunj; P02—Tounj; P03—Istria; P04—Novi Vinodolski; P05—Split; P06—Brač; P07—Konavle. Fruit and seed morphometric characteristics: m (g)—fruit mass; FL (cm)—fruit length; MFW (cm)—maximum fruit width; PMFW (cm)—fruit length, measured from the fruit base to the point of maximum fruit width; FW1 (cm)—fruit width at 15% of fruit length; FW2 (cm)—fruit width at 85% of fruit length; FW/FL—maximum fruit width/fruit length; SL (mm)—seed length; SW (mm)—maximum seed width; NS—number of seeds per fruit.

Population	m		FL		MFW		PMFW		FW1		FW2		FW/FL		SL		SW		NS	
	M	CV	M	CV	M	CV	M	CV	M	CV	M	CV	M	CV	M	CV	M	CV	M	CV
P01	9.53f	27.34	2.37f	10.24	2.40f	11.15	0.99f	17.72	1.91c	12.67	1.56c	16.12	1.02a	9.84	7.26e	9.06	5.51e	10.83	1.35ab	51.85
P02	8,91e	32.22	2.26e	16.03	2.31a	12.89	0.92e	19.30	1.87d	15.67	1.53c	14.22	1.03a	10.75	6.68c	13.66	5.23d	14.33	1.48c	54.40
P03	7.01a	26.03	2.09a	7.36	2.04c	8.92	0.85a	15.32	1.60a	13.15	1.28a	14.71	0.98e	10.41	6.72cd	11.05	4.85a	13.72	1.46bc	37.14
P04	7.49d	31.45	2.15d	11.56	2.31a	12.02	0.85a	21.12	1.92c	13.81	1.46b	17.33	1.08d	6.97	6.39a	9.42	5.07b	10.65	1.25a	47.59
P05	6.86a	30.95	2.06a	11.44	2.21e	12.56	0.88d	18.42	1.76b	15.55	1.46b	15.20	1.07cd	8.53	6.80d	9.54	5.12b	9.59	1.97d	46.18
P06	5.09b	36.12	1.88b	14.96	1.98b	12.43	0.75b	20.90	1.61a	13.46	1.25a	16.14	1.06bc	9.18	6.48ab	10.19	4.42c	14.56	3.66e	25.49
P07	6.34c	28.54	1.99c	11.84	2.09d	10.92	0.78c	19.47	1.75b	12.67	1.32d	18.41	1.06b	11.83	6.53b	13.55	4.87a	14.09	2.00d	56.25
Total	7.32	36.04	2.37	10.24	2.19	13.48	0.86	20.78	1.77	15.62	1.41	18.07	1.04	10.17	6.70	11.71	5.01	14.08	1.88	60.06

**Table 2:** Results of the hierarchical analysis of variance for fruit and seed morphometric characteristics. Acronyms for fruit and seed morphometric characteristics as in Table 1.

Variable	Components of the Variance	df	F	Percent of Variability	*p*-Value
m	Population	6	5.27	25.44	0.000401
	Tree (Population)	42	62.22	41.04	0.000000
	Error			33.52	
FL	Population	6	4.75	22.68	0.000898
	Tree (Population)	42	59.47	41.68	0.000000
	Error			35.64	
MFW	Population	6	5.24	22.49	0.000422
	Tree (Population)	42	45.13	36.34	0.000000
	Error			41.17	
PMFW	Population	6	6.34	16.57	0.000084
	Tree (Population)	42	17.27	20.49	0.000000
	Error			62.94	
FW1	Population	6	4.48	17.66	0.001365
	Tree (Population)	42	37.19	34.57	0.000000
	Error			47.77	
FW2	Population	6	4.50	18.36	0.001325
	Tree (Population)	42	40.08	35.82	0.000000
	Error			45.82	
FW/FL	Population	6	1.32	2.48	0.270911
	Tree (Population)	42	62.51	53.80	0.000000
	Error			43.73	
SL	Population	6	1.52	4.48	0.196602
	Tree (Population)	42	85.93	60.12	0.000000
	Error			35.40	
SW	Population	6	5.20	18.65	0.000445
	Tree (Population)	42	30.35	30.09	0.000000
	Error			51.26	
NS	Population	6	13.27	47.02	0.000000
	Tree (Population)	42	50.24	26.29	0.000000
	Error			26.69	

**Table 3:** Arithmetic means (M) and coefficient of variations (CV/%) for water (g/100 g), crude protein (g/100 g), sugar (g/100 g), ash (g/100 g), crude fat (g/100 g), cellulose (g/100 g), acidity (%), total phenols (mg GAE/g), and antioxidant activity (DPPH %; FRAP mmol Fe^2+^). All mass fractions were determined on a dry mass basis. Different letters within one column denote statistically significant differences (p < 0.05) by ANOVA and Fisher’s LSD test. Acronyms of populations: P01—Psunj; P02—Tounj; P03—Istria; P04—Novi Vinodolski; P05—Split; P06—Brač; P07—Konavle.

Population	Water		Crude Protein		Sugar		Ash		Crude Fat		Cellulose		Acidity		Total Phenols		DPPH		FRAP	
	M	CV	M	CV	M	CV	M	CV	M	CV	M	CV	M	CV	M	CV	M	CV	M	CV
P01	69.50d	2.78	15.32ac	14.47	49.94a	4.81	1.90ab	16.39	0.59d	16.01	5.93ab	10.68	0.74a	13.14	11.11c	63.19	11.99a	10.50	2.41ac	12.58
P02	67.81ad	2.18	12.49b	20.29	49.44a	8.49	2.01ab	44.11	0.60bd	9.22	6.01ab	20.12	0.64a	19.79	12.10c	16.80	4.40b	54.74	2,91bc	1.71
P03	66.52ac	1.59	16.85a	18.61	50.70a	6.95	2.19a	14.23	0.80ac	12.98	5.54ab	6.42	0.70a	8.25	9.44bc	38.23	17.76c	19.42	2.49ab	4.20
P04	67.05a	2.09	12.50b	8.41	55.60c	4.99	1.44b	20.05	0.85c	19.53	5.33ab	8.58	0.68a	10.04	6.57ab	50.33	13.61a	47.36	2.69abc	5.94
P05	64.24bc	3.01	16.67a	14.73	49.47a	3.90	2.29ac	8.09	0.78ac	7.47	5.13a	11.20	0.67a	5.24	5.62a	42.17	13.76a	25.88	2.76bc	12.32
P06	56.80e	6.28	15.63ac	19.56	42.88b	7.66	2.91c	33.90	0.72ab	15.62	9.06c	32.34	0.64a	28.20	3.50a	57.44	11.70a	12.13	2.50ab	12.77
P07	64.05b	3.99	13.41bc	21.96	47.50a	6.83	2.13ab	41.78	0.71ab	22.04	6.65b	10.83	0.67a	11.26	6.82ab	53.65	12.57a	39.97	2.50ab	23.37
Total	65.14	6.70	14.69	20.27	49.36	9.33	2.12	34.23	0.72	19.52	6.24	27.95	0.68	14.90	7.88	57.48	11.81	44.00	2.61	13.34

## Data Availability

Not applicable.

## Supplementary Materials

The following are available online at www.mdpi.com/article/10.3390/plants10081691/s1, Table S1: Environmental variables for seven *Sorbus domestica* populations. Acronyms of populations: P01—Psunj; P02—Tounj; P03—Istria; P04—Novi Vinodolski; P05—Split; P06—Brač; P07—Konavle. Environmental variables: BIO1 = Annual Mean Temperature; BIO2 = Mean Diurnal Range (Mean of monthly (max temp—min temp)); BIO3 = Isothermality (BIO2/BIO7) (×100); BIO4 = Temperature Seasonality (standard deviation ×100); BIO5 = Max Temperature of Warmest Month; BIO6 = Min Temperature of Coldest Month; BIO7 = Temperature Annual Range (BIO5-BIO6); BIO8 = Mean Temperature of Wettest Quarter; BIO9 = Mean Temperature of Driest Quarter; BIO10 = Mean Temperature of Warmest Quarter; BIO11 = Mean Temperature of Coldest Quarter; BIO12 = Annual Precipitation; BIO13 = Precipitation of Wettest Month; BIO14 = Precipitation of Driest Month; BIO15 = Precipitation Seasonality (Coefficient of Variation); BIO16 = Precipitation of Wettest Quarter; BIO17 = Precipitation of Driest Quarter; BIO18 = Precipitation of Warmest Quarter; BIO19 = Precipitation of Coldest Quarter, Figure S1: Biplot of the principal component analysis based on 19 bioclimatic variables in seven studied *Sorbus domestica* populations. Acronyms of populations: P01 (Psunj), P02 (Tounj), P03 (Istria), P04 (Novi Vinodolski), P05 (Split), P06 (Brač), and P07 (Konavle). Acronyms for environmental variables as in Table S1, Table S2: Pearson’s correlation coefficients between 19 bioclimatic variables and scores of the first three principal components. Acronyms for environmental variables as in Table S1, Figure S2: *Sorbus domestica* fruit samples from seven studied populations, Table S3: Pearson’s correlation coefficients between ten morphological traits and scores of the first three principal components, Table S4: Pearson’s correlation coefficients between ten chemical traits and scores of the first three principal components, Table S5: Results of the stepwise discriminant analyses for morphometric traits. *p*(λ), significance of Wilks’λ: *** significant at *p* < 0.001, ** significant at 0.001 < *p* < 0.01, * significant at 0.01 < *p* < 0.05, ns non-significant values (*p* > 0.05), Table S6: Results of the stepwise discriminant analyses for chemical traits. *p*(λ), significance of Wilks’λ: *** significant at *p* < 0.001, ** significant at 0.001 < *p* < 0.01, * significant at 0.01 < *p* < 0.05, ns non-significant values (*p* > 0.05).
