# Supplementary material for: Morphological and Chemical Diversity and Antioxidant Capacity of the Service Tree (Sorbus domestica L.) Fruits from Two Eco-Geographical Regions
Source: Plants (Basel). 2021 Aug 17;10(8):1691. doi: 10.3390/plants10081691 (PMC8399663; doi:10.3390/plants10081691)
Supplement: Supplementary file 1 [file plants-10-01691-s001.zip › plants-1329376-supplementary.pdf]

Table S1. Environmental variables for seven *Sorbus domestica* populations. Acronyms of populations: P01 – Psunj; P02 – Tounj; P03 – Istria; P04 – Novi Vinodolski; P05 – Split; P06 – Brač; P07 – Konavle. Environmental variables: BIO1 = Annual Mean Temperature; BIO2 = Mean Diurnal Range (Mean of monthly (max temp - min temp)); BIO3 = Isothermality (BIO2/BIO7) (×100); BIO4 = Temperature Seasonality (standard deviation ×100); BIO5 = Max Temperature of Warmest Month; BIO6 = Min Temperature of Coldest Month; BIO7 = Temperature Annual Range (BIO5-BIO6); BIO8 = Mean Temperature of Wettest Quarter; BIO9 = Mean Temperature of Driest Quarter; BIO10 = Mean Temperature of Warmest Quarter; BIO11 = Mean Temperature of Coldest Quarter; BIO12 = Annual Precipitation; BIO13 = Precipitation of Wettest Month; BIO14 = Precipitation of Driest Month; BIO15 = Precipitation Seasonality (Coefficient of Variation); BIO16 = Precipitation of Wettest Quarter; BIO17 = Precipitation of Driest Quarter; BIO18 = Precipitation of Warmest Quarter; BIO19 = Precipitation of Coldest Quarter.

Pop.	bio1	bio2	bio3	bio4	bio5	bio6	bio7	bio8	bio9	bio10	bio11	bio12	bio13	bio14	bio15	bio16	bio17	bio18	bio19
P01	10.8	9.4	31.9	716.8	24.5	-5.0	29.5	19.5	3.2	19.5	1.8	952	103	54	17.8	281	178	281	186
P02	10.4	10.8	34.6	730.5	24.8	-6.5	31.3	10.7	2.8	19.2	1.1	1255	136	75	18.5	391	242	308	258
P03	12.8	9.2	32.7	707.5	26.5	-1.7	28.2	13.1	5.4	21.8	4.4	1042	116	60	20.2	327	208	248	228
P04	13.6	7.3	29.0	670.6	26.6	1.3	25.3	14.0	22.3	22.3	5.9	1211	155	57	32.1	450	228	228	280
P05	13.6	8.5	31.4	695.7	27.9	0.9	27.0	9.9	22.6	22.6	5.6	827	107	34	28.4	286	138	138	223
P06	15.3	7.0	27.6	690.4	28.0	2.8	25.2	11.7	24.2	24.2	7.3	777	102	29	31.2	275	119	119	221
P07	15.8	7.3	30.6	621.1	28.1	4.2	23.9	13.0	23.8	23.8	8.8	1254	183	29	46.1	497	127	127	423

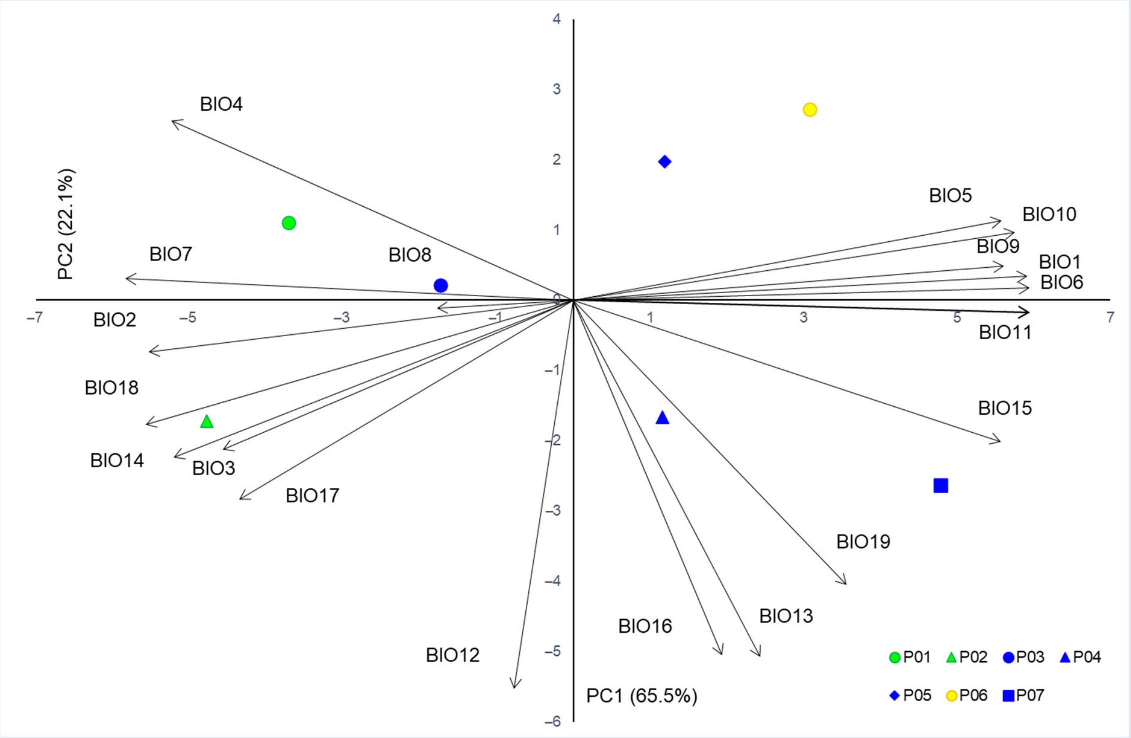

Figure S1. Biplot of the principal component analysis based on 19 bioclimatic variables in seven studied *Sorbus domestica* populations. Acronyms of populations: P01 (Psunj), P02 (Tounj), P03 (Istria), P04 (Novi Vinodolski), P05 (Split), P06 (Brač), and P07 (Konavle). Acronyms for environmental variables as in Table S1.

Table S2. Pearson's correlation coefficients between 19 bioclimatic variables and scores of the first three principal components. Acronyms for environmental variables as in Table S1.

Environmental variables	PC—Principal Component		
	PC1	PC2	PC3
BIO1	0.985817	0.041339	-0.044234
BIO2	-0.922173	-0.122140	-0.288829
BIO3	-0.761164	-0.321878	-0.383230
BIO4	-0.872703	0.426822	-0.166406
BIO5	0.930220	0.172392	-0.294272
BIO6	0.990758	0.030378	0.007727
BIO7	-0.972323	0.052270	-0.179615
BIO8	-0.295793	-0.001770	0.927866
BIO9	0.934964	0.082226	-0.065905
BIO10	0.958224	0.160897	-0.093932
BIO11	0.990957	-0.028667	-0.017851
BIO12	-0.128451	-0.989237	-0.001729
BIO13	0.405168	-0.911966	0.017329
BIO14	-0.868277	-0.372458	-0.040242
BIO15	0.928836	-0.336795	0.016881
BIO16	0.323219	-0.942486	0.019305
BIO17	-0.726478	-0.472929	-0.016479
BIO18	-0.929885	-0.294792	0.165404
BIO19	0.593301	-0.758317	-0.078756
Eigenvalue	12.46362	4.205575	1.289686
% Total Variance	65.59799	22.13461	6.787819

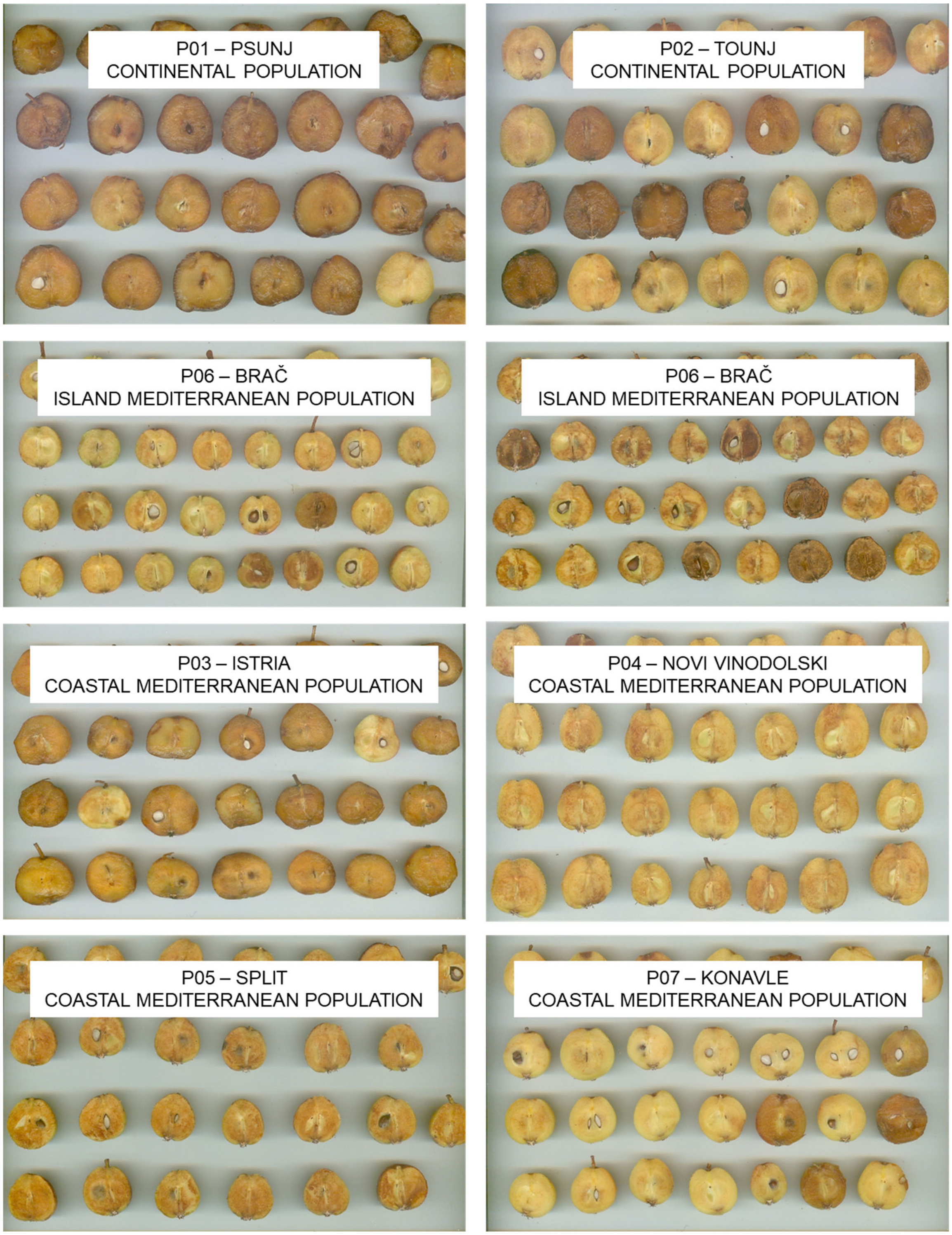

Figure S2. *Sorbus domestica* fruit samples from seven studied populations.

Table S3. Pearson's correlation coefficients between ten morphological traits and scores of the first three principal components.

Trait	PC—Principal Component		
	PC1	PC2	PC3
Fruit Mass	-0.927652	-0.034356	0.106469
Fruit Length	-0.899328	0.285453	0.258839
Maximum Fruit Width	-0.907215	-0.384038	0.098813
Position of Maximum Fruit Width	-0.883715	0.189022	0.187832
Fruit Width 1	-0.811658	-0.465808	0.108048
Fruit Width 2	-0.807114	-0.517059	-0.019728
Maximum Fruit Width/Fruit Length	0.132294	-0.938399	-0.233767
Seed Length	-0.612588	0.540219	0.054570
Seed Width	-0.789985	0.131612	-0.462564
Number of Seeds	0.565801	-0.340882	0.632544
Eigenvalue	5.920514	2.056156	0.807143
% Total Variance	59.20514	20.56156	8.07143

Table S4. Pearson's correlation coefficients between ten chemical traits and scores of the first three principal components.

Trait	PC—Principal Component		
	PC1	PC2	PC3
Water	-0.875312	0.116830	0.286291
Crude proteins	0.153992	-0.484624	0.069657
Sugars	-0.824995	-0.320994	0.064157
Ash	0.755234	0.090704	0.259989
Crude Fat	-0.118221	-0.682250	-0.195699
Cellulose	0.776374	0.302306	-0.057561
Acidity	0.092736	0.105715	0.781612
DPPH	0.093218	-0.774208	0.210610
FRAP	-0.377734	0.374581	-0.516128
Total Phenols	-0.540674	0.495550	0.312717
Eigenvalue	3.109915	1.913083	1.219590
% Total Variance	31.09915	19.13083	12.19590

Table S5. Results of the stepwise discriminant analyses for morphometric traits. p(λ), significance of Wilks'λ: *** significant at p< 0.001, ** significant at 0.001 <p< 0.01, * significant at 0.01 <p< 0.05, ns non-significant values(p> 0.05).

Trait	Wilks' Lambda	Partial Lambda	F-Remove	p-Value
Fruit Mass	0.181243	0.966230	0.664055	ns
Fruit Length	0.227957	0.768225	5.732343	**
Maximum Fruit Width	0.185318	0.944985	1.106131	ns
Position of Maximum Fruit Width	0.219460	0.797971	4.810396	*
Fruit Width 1	0.213889	0.818753	4.206012	*
Fruit Width 2	0.200534	0.873283	2.756981	ns
Seed Length	0.194915	0.898457	2.147363	ns
Seed Width	0.190265	0.920414	1.642887	ns
Number of Seeds	0.299037	0.585623	13.444110	***

Table S6. Results of the stepwise discriminant analyses for chemical traits. p(λ), significance of Wilks'λ: *** significant at p< 0.001, ** significant at 0.001 <p< 0.01, * significant at 0.01 <p< 0.05, ns non-significant values(p> 0.05).

Trait	Wiks' Lambda	Partial Lambda	F-Remove	p-Value
Water	0.149932	0.577806	13.517690	***
Crude Proteins	0.089422	0.968793	0.595925	ns
Sugars	0.090458	0.957693	0.817260	ns
Ash	0.087305	0.992287	0.143804	ns
Crude Fat	0.118228	0.732748	6.747410	**
Cellulose	0.101290	0.855285	3.130210	*
Acidity	0.089834	0.964351	0.683881	ns
Total Phenols	0.090239	0.960018	0.770467	ns
DPPH	0.102515	0.845058	3.391977	*
FRAP	0.088468	0.979236	0.392285	ns
